# Small-Molecule Probes: Recent Progress in the Rapid Detection of Carbapenemase-Producing Bacteria

**DOI:** 10.3390/pharmaceutics17030282

**Published:** 2025-02-20

**Authors:** Feiran Xie, Yanzhi Zhou, Fei Zhang, Peihong Xiao

**Affiliations:** 1 International Medical College, Chongqing Medical University, Chongqing 401331, China; fx26@student.le.ac.uk; 2 Department of Laboratory Medicine and Sichuan Provincial Key Laboratory for Human Disease Gene Study, Sichuan Provincial People’s Hospital, School of Medicine, University of Electronic Science and Technology of China, Chengdu 610041, China; 3 Hubei Key Laboratory of Radiation Chemistry and Functional Materials, School of Nuclear Technology and Chemistry & Biology, Hubei University of Science and Technology, Xianning 437000, China; zhangfei@hbust.edu.cn

**Keywords:** small-molecule probes, carbapenemase-producing bacteria (CPB), multidrug-resistant bacteria, rapid detection

## Abstract

As the last resort and one of the most crucial antibiotics for multidrug-resistant bacteria, carbapenem is considered the best hope for treating bacterial infections. However, the prompt emergence of carbapenemase-producing bacteria (CPB) poses a striking global health threat. Thus, accurate and rapid methods for the detection of carbapenemase are being requested to guide precise diagnosis, appropriate treatment strategies, and antibiotic stewardship. Although genotypic, phenotypic, and biochemical methods are currently used in clinical practice for CPB detection, they each have their problems that cannot commendably meet the need. In recent years, small-molecule probes have made significant progress and breakthroughs in the rapid detection and subtyping of CPB, providing insights and innovative solutions for the ultra-sensitive detection of CPB. In this minireview, some of the advances, namely, chromogenic probes and methods, fluorogenic probes, dual fluorogenic–chromogenic probes, a chemiluminescent probe, and a novel label-free intracellular calorimetric approach, are summarized, appreciated, and discussed. These methods offer high sensitivity, specificity, and accuracy in a short period in clinical settings without the utilization of sophisticated equipment or professional personnel. We hope that this minireview can provide a reference for the development of rapid detection of CPB and eventually contribute to antibiotic resistance management.

## 1. Introduction

As an atypical *β*-lactam antibiotic, carbapenem holds the broadest spectrum and the most prominent activity against bacteria, and it has long been viewed as the last bactericidal for treating serious bacterial infections caused by multidrug-resistant bacteria due to its low toxicity and stability toward *β*-lactamases. Typical carbapenems include imipenem, meropenem, doripenem, and ertapenem, etc. [1]. Carbapenemase, a compelling *β*-lactamase, includes a group of enzymes capable of hydrolyzing virtually all the *β*-lactam antimicrobial drugs, including carbapenem, by cleaving the *β*-lactam ring, thus obstructing antimicrobial efficacy [2]. According to the Ambler classification system, carbapenemases are mainly divided into three groups based on their amino acid identity. Class A (e.g., KPC) and class D (e.g., OXA-48) are so-called serine *β*-lactamases that form an acyl-enzyme intermediate when catalyzing carbapenems. Class B, also known as the metallo-*β*-lactamases (MBLs), depends on zinc to initiate the hydrolytic reaction. It includes the imipenem enzyme-producing *β*-lactamase (IMP), Verona-integron encoded metallo-*β*-lactamase (VIM), and New Delhi metallo-*β*-lactamase (NDM-1) [3]. In recent years, the emergence and dissemination of antibiotic resistance among bacteria has posed an escalating global health threat. Among various mechanisms accounting for antibiotic resistance, the production of carbapenemases by bacteria has become a major concern [4]. Most frequently, carbapenemases are encoded on mobile genetic components like plasmids that can be easily transferred across species, including Enterobacteriaceae, through horizontal gene transfer [5]. The alarming rise of antibiotic resistance, especially in CPB, has presented a grave challenge to healthcare systems worldwide, as it severely limits the effectiveness of existing antibiotics and hinders the progress of modern medicine. The rapid and accurate detection of carbapenemase in bacteria is crucial for guiding clinical antibiotic therapy and standardizing its stewardship to prevent the further spread of antibiotic resistance [6].

Existing detection methods for carbapenemase in clinical practice can be mainly classified into three categories based on the underlying principles and techniques utilized, namely, genotypic, phenotypic, and biochemical methods. Table 1 provides a summary of the detection time, cost, advantages, and limitations of several commonly used detection methods in clinical practice for carbapenemases. Polymerase Chain Reaction (PCR) and real-time quantitative reverse transcriptase PCR (rt qRT-PCR) are traditional genotypic methods to detect the presence of specific genes associated with carbapenemase [7,8]. Genotypic methods provide the highest sensitivity and specificity when subtyping carbapenemases and offer clues to exploring antibiotic resistance genes. However, only known, targeted genes can be detected, leaving unknown or rare genes undiscovered [9]. Furthermore, delicate equipment and trained staff are largely required, making it less practical for daily screening and impractical to promote in resource-limited areas [10]. Phenotypic methods like the modified Hodge test (MHT), Carba NP test, Boronic Acid Disk Test, the Epsilometer Test (E-test), and the double disc synergy test (DDST), examining carbapenem susceptibility or carbapenemase activity, are relatively uncomplicated and cost-effective, ready to perform in most clinical laboratories [11,12,13,14,15]. Still, phenotypic methods lack specificity, as other factors like porin mutations or efflux pumps, which reduce carbapenem susceptibility, may interfere with the actual result [16]. In addition to these, they are also typically time-consuming and labor-intensive. Biochemical methods such as Mass Spectrometry (MS), together with Liquid Chromatography–Tandem Mass Spectrometry (LC-MS/MS) and Matrix-assisted Laser Desorption/Ionization Time-of-Flight Mass Spectrometry (MALDI-TOF MS), identify and quantify carbapenemase activity using the mass change from carbapenem hydrolysis [17,18,19]. Rapid and sensitive though they are, sophisticated instrumentation is demanded, and professionals are needed to interpret the result. Availability in routine clinical laboratories may be restricted. Therefore, there is an urgent need for the development and implementation of assays that are rapid, reliable, operationally simple, and economical for the detection of CPB.

**Figure 1:**
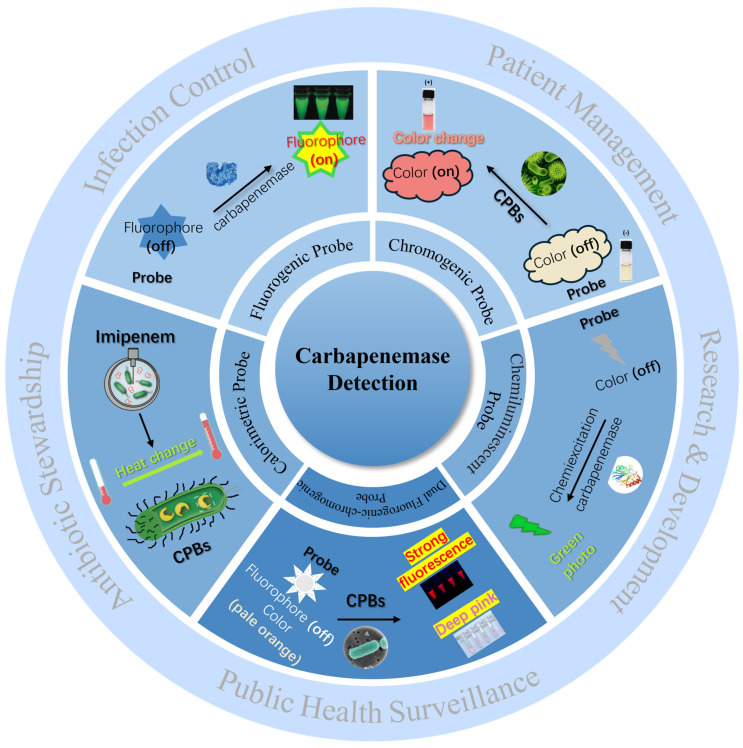
Schematic illustration of small-molecule probes in carbapenemase detection and their applications.

Recently reported methods based on small-molecule probes for CPB detection have gained much attention. In this review, several methods that can detect CPB with rapidity, reliability, simplicity, and cost-effectiveness are mentioned to aid in implementing effective infection control measures, preventing the spread of resistance, and improving patient outcomes, ultimately contributing to the fight against antibiotic resistance for public health protection. In this minireview, we first provide a comprehensive summary of the advantages and limitations of current clinical detection methods for carbapenemase. Secondly, the research progress of detection methods based on small-molecule probes, including chromogenic probes, fluorogenic probes, dual fluorogenic–chromogenic probes, and chemiluminescent probes, is highlighted (Figure 1). Finally, the challenges faced by these reported probes in practical clinical applications, new insights into molecular probe design, and future opportunities in this research field are provided in the last section.

## 2. Chromogenic Methods in Carbapenemase Detection

Chromogenic probes have the advantages of simple operation, cost-effectiveness, and visualized results, and they have aroused the research interest of clinical and scientific researchers over the past decades. A widely acknowledged approach based on enzymatic hydrolysis of nitrocefin, a chromogenic substrate, rapidly reports the presence of *β*-lactamase. At the same time, the traditional Carba NP assay is the only carbapenemase-specific chromogenic test that has been recommended by the Clinical and Laboratory Standards Institute (CLSI) of the US [20,21]. According to the CLSI, the principle of Carba NP is that phenol red displays a distinct color change from yellow to red in 2 h upon hydrolysis of *β*-lactam rings from imipenem into carboxylic acid by carbapenemase, indicating the presence of CPB. However, the acidity of the solution should reach a threshold to allow the color change to be visible by the naked eye, resulting in low sensitivity, with potential false negative results frequently appearing. Moreover, it remains a challenge to subtype carbapenemases by using the aforementioned methods. In 2019, Hobbs et al. established a Nitro-Carba test based on the hydrolysis of nitrocefin by carbapenemase in the presence of carbapenem antibiotics to distinguish it from other *β*-lactamases [22]. In the context of carbapenem antibiotics, imipenem is known to inhibit the majority of *β*-lactamases interacting with nitrocefin. Similarly, meropenem hinders the ability of AmpC *β*-lactamases to hydrolyze nitrocefin. Additionally, ertapenem is found to effectively inhibit ESBLs, AmpC *β*-lactamases, and co-produced ESBLs and AmpC *β*-lactamases to hydrolyze nitrocefin. Therefore, after nitrocefin is added, a color change from yellow to red is observed within 20 min to indicate the presence of carbapenemase, while the color change does not appear without the activity of carbapenemase or *β*-lactamases.

**Figure 2:**
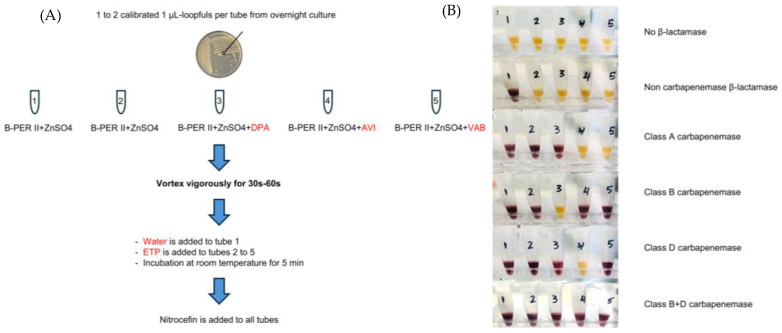
(**A**) Schematic diagram of the experimental process. (**B**) Experimental results and the corresponding interpretation. Adapted with permission from ref. [23]. Copyright 2020, America Society for Microbiology.

Although the Nitro-Carba test modified the Carba NP test and offered a simple, responsive, and cost-effective strategy in a much shorter time, it still could not realize carbapenemase subtyping. As a derivative method of the Nitro-Carba test, the NitroSpeed-Carba NP test, a biochemical test recently established by Nordmann and co-workers, overcame this barrier, effectively recognizing the majority of types of carbapenemases, even OXA-like carbapenemases and other potential novel carbapenemases, which were hardly detected by other methods due to low enzyme activity or low corresponding genetic expression level [23]. As illustrated in the protocol, nitrocefin was added to a series of tubes, some without special management and others with carbapenemase-specific inhibitors, namely dipicolinic acid (class B inhibitor), avibactam (class D inhibitor), and vaborbactam (class A inhibitor), respectively. The results were interpreted to identify carbapenemase activity and subtypes according to the mode of color change in each tube in less than 15 min (Figure 2A,B). During a screening test, 248 genetically characterized clinical Enterobacterales isolates from multiple sources, including blood cultures, urine, and sputum, were tested, where various types of Enterobacterales and subtypes of CPB (even class D and co-producing strains) were included. The overall sensitivity (100%) and specificity (97.1%) were confirmed. The only three false positive results were from ACC or CMY AmpC types, which are known to possess low enzyme activity. Under complex biological environments, such as blood and urine samples with fluctuating pH levels or protein and/or glucose levels, testing results might be altered. However, this clinical screening confirmed that the NitroSpeed-Carba NP test could significantly avoid biological interference.

**Figure 3:**
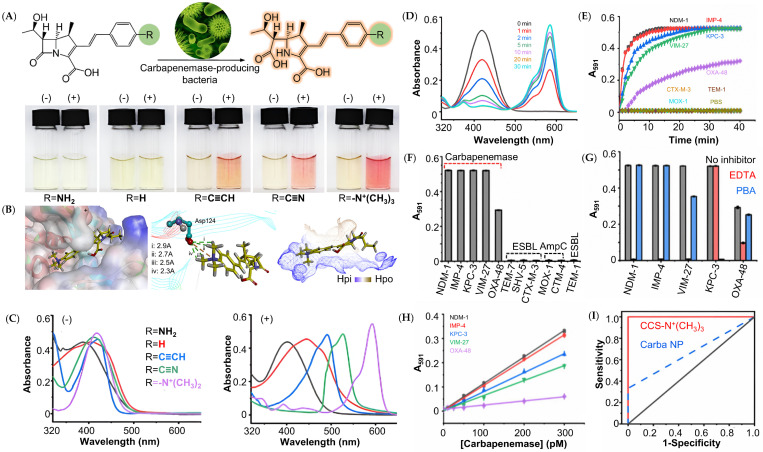
(**A**) Schematic representation of the cleavage of the *β*-lactam ring of CCS with different R substituent groups by carbapenemase and their color changes after incubation with NDM-1. (**B**) The electrostatic force, van der Waals force, hydrogen bonds, and hydrophobic interaction between CCSs and NDM-1 (“+”: electrostatic surface potentials). (**C**) The UV/Vis spectra of CCSs after incubation with NDM-1. (**D**) The UV/Vis spectra of CCS-N^+^(CH_3_)_3_ after incubation with NDM-1 for 30 min. (**E**) Time-dependent absorbance changes at 591 nm of CCS-N^+^(CH_3_)_3_ after incubation with carbapenemases and non-carbapenemases. (**F**) A_591_ of CCS-N^+^(CH_3_)_3_ after incubation with a range of *β*-lactamases. (**G**) A_591_ of CCS-N^+^(CH_3_)_3_ after incubation with five carbapenemases with and without the presence of inhibitors. (**H**) A_591_ of CCS-N^+^(CH_3_)_3_ after incubation with five carbapenemases in a concentration-dependent manner. (**I**) The Receiver Operating Characteristic (ROC) Curve of the Carba NP test and CCS-N^+^(CH_3_)_3_-based POC method. Adapted with permission from ref. [25]. Copyright 2022, American Chemical Society.

Small-molecule chromogenic probes are engineered to generate a colorimetric signal upon interaction with carbapenemase enzymes, enabling the direct visual detection of CPB by the naked eye in clinical samples. These probes generally comprise a carbapenem core linked to a chromophore, which undergoes a color change upon cleavage by carbapenemase to allow the straightforward and rapid identification of CPB [24]. Lately, Liu et al. described the first ultra-sensitive chromogenic probe based on the chromogenic carbapenem substrate (CCS) that instantly identified the existence of CPB in clinical sputum by a striking color change from yellow to red within 15 min, accomplishing the task of real-time detection of carbapenemase [25]. The probe adopted a three-building-block design that consisted of a carbapenem core, a benzene derivative, and a terminated para-substituent with different electronegativities; it was proven later that the sensitivity of the probe was proportional to the electron-withdrawing capability of the substituent (Figure 3A,B). For instance, a substituent with strong electron-withdrawing ability showed a detection limit of three orders of magnitude lower than that of the Carba NP test, being the first chromogenic probe to easily detect carbapenemase at the picomolar level. One explanation for this that the team discovered was that positively charged substituents like -N^+^(CH_3_)_3_ enhanced the molecular affinity of the probe to carbapenemases through stable electrostatic interactions, thus improving its sensitivity. To our delight, CCS-N^+^(CH_3_)_3_ allowed visualization of OXA-48, providing ultra-sensitivity (Figure 3C–E). Furthermore, CCS-N^+^(CH_3_)_3_ also exhibited more outperforming chemical stability than other, similar carbapenemase probes. Selectivity was determined by the inhibitory test, while the sensitivity and the specificity of the probe were tested with both carbapenemases and commonly seen CPB isolates in clinical scenarios in a series of concentrations (Figure 3F–H). The original aqueous solution of CCS-N^+^(CH_3_)_3_ was integrated into a paper-based device for point-of-care testing (POCT). Therefore, it offered an affordable, portable, and versatile diagnostic solution, the benefits of which included minimal sample volume requirements, rapid results, and biodegradability, making it ideal for resource-limited settings and remote healthcare delivery [26]. Impressively, 80 sputum samples collected from sepsis patients with lung infections (mostly infected by antibiotic-resistant bacteria) were screened using CCS-N^+^(CH_3_)_3_ applied paper clips, which gave 100% sensitivity and specificity. The bacteria included five types of CPB characterized by rt-PCR. Furthermore, a color recognition app was set up on smartphones to directly read and quantify the visual results without any other special processing, and it also showed 100% correct readout. Compared with common detection methods, CCS-N^+^(CH_3_)_3_ demonstrated much better sensitivity and speed than a series of classical Carba NP tests or mCIM and better simplicity, cost, and rapidity than rt-PCR (Figure 3I). In contrast with fluorogenic probes reported in previous articles, CCS-based chromogenic probes were constructed with simpler structures but with high stability and flexibility. Moreover, by finely tuning the carbapenem core, it is expected to rapidly detect *β*-lactamases other than carbapenemases, contributing to wider and more integrated antibiotic stewardship and infection control.

Chromogenic probes yield rapid results, facilitating the rapid detection of CPB in clinical samples. Additionally, these probes can be readily adapted for use in resource-limited settings where more complex detection methods may not be feasible. Despite these advantages, the limitations include that the sensitivity and specificity may not bear comparison with those of more advanced techniques such as PCR [27]. Additionally, some chromogenic probes are not capable of identifying all types of carbapenemase enzymes, as their effectiveness relies on specific interactions between the probe and the enzyme [20,21,22]. Nevertheless, chromogenic probes represent a valuable tool for detecting CPB, particularly in settings where sophisticated detection methods are unavailable.

## 3. Fluorescent Probes in Carbapenemase Detection

Fluorescent probes are widely applied in various fields, including biology and medicine, for detecting and imaging specific targets like enzymes or biomolecules in living cells or tissues [28]. By exploiting the enzymatic activity of carbapenemase, fluorescent probes are also adopted in the field of rapid carbapenemase detection in bacteria, where they are designed to mimic a structure similar to that of a carbapenem antibiotic but with an attached fluorophore. Once encountering a sample containing carbapenemase, the probe will be cleaved and release the fluorophore, which leads to fluorescence emission that can then be visualized and quantified, providing a rapid and sensitive result [29].

**Figure 4:**
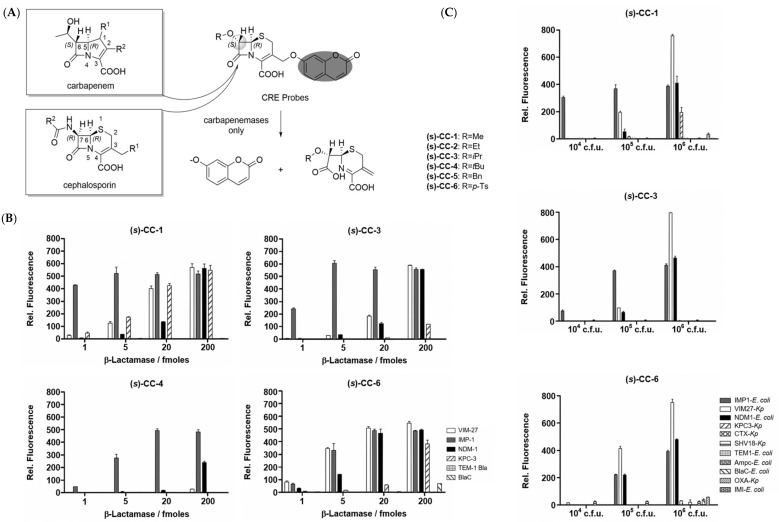
(**A**) The structure of carbapenem-based CRE probe. (**B**) The relative fluorescence of the probes upon incubation with a series of *β*-lactamases. (**C**) The relative fluorescence of the probes upon incubation with a series of bacteria-producing *β*-lactamases. Adapted with permission from ref. [30]. Copyright 2014, Wiley Online Library.

Through a close investigation into the structure of carbapenem and other *β*-lactams, Rao et al. first synthesized a series of fluorogenic probes based on stereo-chemically modified cephalosporin that were specifically tailored to detect carbapenem-resistant Enterobacteriaceae (CRE) successfully in both recombinant enzymes and live bacterial species experiments in 2014 [30]. Cephalosporin, a typical *β*-lactam, is characterized by an R configuration at both C6 and C7, while carbapenem, having good resistance to general *β*-lactamases, possesses a trans configuration in the *β*-lactam ring (R configuration at C5 and S configuration at C6) [31]. Drawing inspiration from the structure of carbapenems, they ingeniously reversed the original cis configuration at the C7 position of cephalosporin, which serves as the enzyme recognition site, and conjugated it with caged umbelliferone at the C3 position, acting as the fluorophore. The R group was changed according to the substrate preference of different types of carbapenemase, modulating their selectivity (Figure 4A). As a result, they found that compounds (S)-CC-1 and (S)-CC-5 were specific to three class B *β*-lactamase (VIM-27, IMP-1, NDM-1) and one class A *β*-lactamase (KPC-3), while (S)-CC-6 was a more sensitive indicator for the former ones. Noticeably, (S)-CC-4 exhibited high specificity and kinetic efficiency for IMP-1, which was of significant importance since MBLs especially lack susceptibility to existing inhibitors, as they frequently transmit across strains (Figure 4B,C) [32].

**Figure 5:**
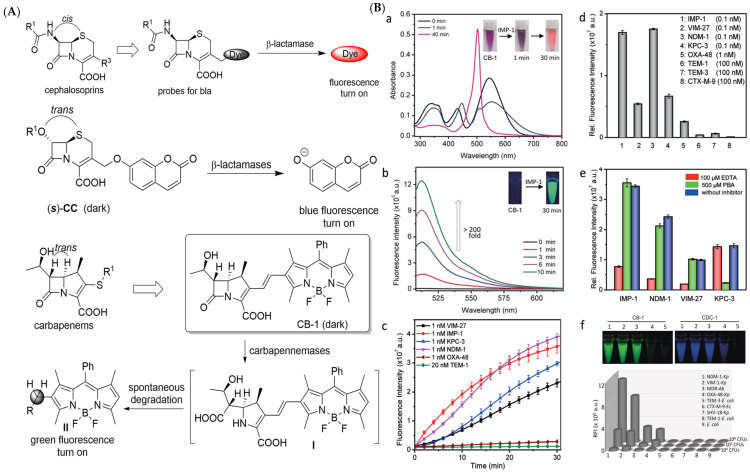
(**A**) Design of carbapenemase-specific fluorogenic probes. (**B**) Sensitivity and specificity of CB-1. (**a**) The UV/Vis spectra of CB-1 after incubation with IMP-1. (**b**) The fluorescence intensity spectra of CB-1 after incubation with IMP-1. (**c**) Time-dependent fluorescence intensity of CB-1 upon incubation with a series of *β*-lactamases. (**d**) The fluorescence intensity changes in CB-1 after incubation with a series of *β*-lactamases. (**e**) The relative fluorescence intensity of CB-1 upon incubation with four carbapenemases with and without the presence of inhibitors. (**f**) Comparison of the relative fluorescence intensities of CB-1 and CDC-1 upon incubation with various bacteria producing *β*-lactamases in concentrations of three adjacent orders of magnitude. Adapted with permission from ref. [33]. Copyright 2017, Wiley Online Library.

However, most *β*-lactamase probes based on cephalosporin were relatively unstable and readily hydrolyzed by common *β*-lactamases in clinical settings. Therefore, another study, led by Xie and his team, developed a carbapenem-derived fluorogenic probe, CB-1, to directly enhance the specificity of carbapenemase detection [33]. Fluorogenic probes can be easily degraded, or they undergo quick photobleaching due to their unique chemical structure. However, CB-1 was relatively more stable than previous cephalosporin-based probes. They chose an alkenyl-linked boron dipyrromethene dye (BODIPY) as the fluorophore, which remained non-fluorescent initially. A carbapenem core was conjugated with BODIPY, and when the core structure was hydrolyzed by carbapenemase, the probe would generate the labile dienamine I, which underwent further spontaneous isomerization or degradation processes that disrupted the conjugation between carbapenem and BODIPY, leading to a strong green fluorescence signal, which demonstrated an impressive selectivity toward carbapenemases (limited concentration of 100 nM) over other prevalent *β*-lactamases (Figure 5A). Notable spectral shifts were seen against IMP-1 (Figure 5B(a,b)), and distinct fluorescence intensity was obtained among carbapenemases rather than other *β*-lactamases (Figure 5B(c,d)). Its specificity was validated through the inhibitory test (Figure 5B(e)), though CDC-1 seemed more sensitive in detecting the OXA-48 type of carbapenemase than CB-1 (Figure 5B(f)). Additionally, CB-1 showed exact results in carbapenemase-producing organisms (CPOs) screening that distinguished the CPOs from other antibiotic-resistant bacteria as expected.

**Figure 6:**
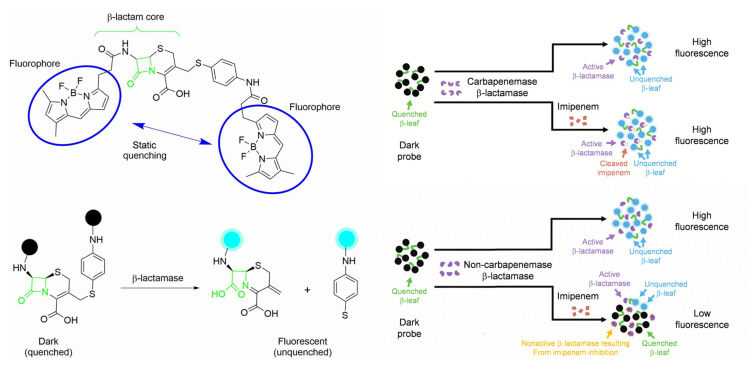
Schematic demonstration of the guideline of FIBA to turn on fluorescence recognition of *β*-lactamase activity and the identification of carbapenemase. Adapted with permission from ref. [34]. Copyright 2020, U.S. Department of Health and Human Services, Centers for Disease Control and Prevention.

To our delight, a novel fluorescent probe, FIBA, has been developed recently to achieve rapid detection of carbapenemases toward 76 clinically important isolates covering 8 major epidemic carbapenemase enzyme types from 16 species in 10 min with 100% sensitivity and specificity in general, requiring only a single mixing step [34]. The dark fluorescence probe consists of a *β*-lactamase core and two primarily quenched *β*-LEAFs (*β*-lactamase enzyme-activated fluorophores), which would emit a fluorescent signal upon cleavage by carbapenemase. To differentiate carbapenemases from other *β*-lactamases, imipenem was added, and CPB exhibited high fluorescence, while non-CPBs showed a slow increase rate in fluorescence since imipenem competed to occupy the receptors bound by the probe. Most critically, the FIBA method was extensible, allowing the use of different inhibitors instead of imipenem to discriminate between various types of carbapenemases (Figure 6).

**Figure 7:**
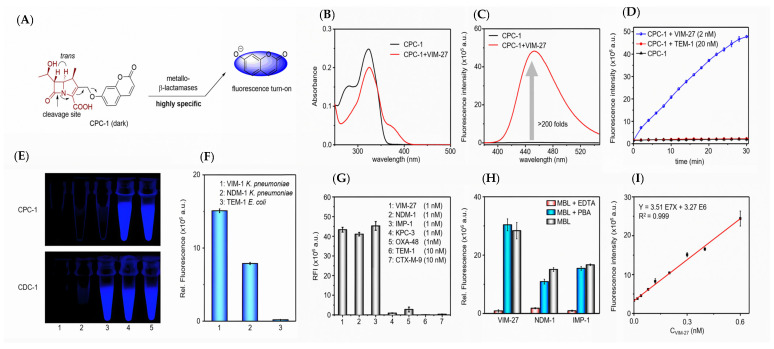
(**A**) The structure and fluorescence turn-on mechanism of CPC-1, a highly MBLs-specific probe. (**B**) The UV/Vis spectra of CPC-1 after incubation with VIM-27. (**C**) The fluorescence intensity spectra of CPC-1 after incubation with VIM-27. (**D**) Time-dependent fluorescence intensity of CPC-1 after incubation with VIM-27 and TEM-1. (**E**) Imaging of fluorescence turn-on of CPC-1 and CDC-1 after incubation with various *β*-lactamase-producing *E. coli* (tube 3 contains TEM-1-transformed *E. coli*). (**F**) The relative fluorescence of CPC-1 after incubation with three *β*-lactamase-encoded antibiotic-resistant bacteria that are significant in clinics. (**G**) The relative fluorescence intensity of CPC-1 after incubation with a series of *β*-lactamases. (**H**) The relative fluorescence of CPC-1 after incubation with VIM-27, NDM-1, and IMP-1 with and without the presence of inhibitors. (**I**) A linear correlation between the concentration of VIM-27 and the fluorescent intensity of CPC-1. Adapted with permission from ref. [35]. Copyright 2018, Wiley-VCH Verlag GmbH & Co. KGaA.

Ambler B *β*-lactamases are a type of carbapenemases that depend on metal ions, including IMP, VIM, and NDM-*β*-lactamases. Xie et al. chose umbelliferone as the activatable fluorophore and adopted carbapenem as the enzyme recognition moiety to construct CPC-1, a carbapenemase-specific fluorogenic probe. The probe could be activated by carbapenemases, typically MBLs, to turn on fluorescence from umbelliferone (Figure 7A) [35]. CPC-1 demonstrated good chemical stability in phosphate-buffered saline (PBS), with a relatively slow spontaneous hydrolysis rate of 2.5 × 10^−6^ s^−1^, giving it an estimated half-life of around 78 h. The hydrolysis of carbapenemases to CPC-1 and the fluorescence spectrum of CPC-1 were confirmed using VIM-27 as an example (Figure 7B,C). Upon incubation with a series of different types of *β*-lactamases, CPC-1 showed a quick and significant fluorescence signal (>200-fold) with MBLs (VIM-27, NDM-1, and IMP-1) in a concentration-dependent manner, while others (TEM-1, KPC-3, OXA-48, and CTX-M-9) exhibited poor or no signals, indicating high specificity toward MBLs. Hydrolysis of CPC-1 by MBLs was further proved by incubating with and without inhibitors (Figure 7D–I).

The advantages of fluorescent probes for detecting carbapenemase in bacteria include high sensitivity and specificity, short detection times, and exceptional visualization of the presence of the enzyme in clinical samples. Fluorescent probes reach simple and rapid identification of carbapenemases while ensuring higher accuracy than colorimetric methods.

## 4. A Dual Fluorogenic–Chromogenic Probe in Carbapenemase Detection

**Figure 8:**
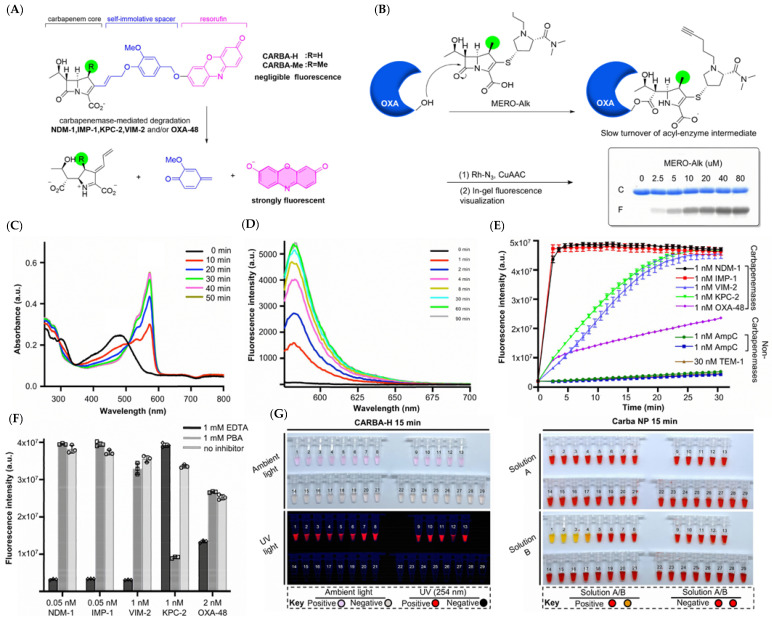
(**A**) The structure of CARBA-H and CARBA-Me and their schematic process of recognizing carbapenemases. (**B**) CuAAC and SDS-PAGE analysis of MERO-Alk upon incubation with OXA-48. (**C**) The absorbance changes of CARBA-H upon incubation with IMP-1 in 50 min. (**D**) The change in fluorescence intensity of CARBA-H upon incubation with IMP-1 in 90 min. (**E**) The time-dependent fluorescence intensity changes of CARBA-H upon incubation with a range of carbapenemases and non-carbapenemases. (**F**) The fluorescence intensity of CARBA-H upon incubation with five carbapenemases with and without the presence of inhibitors. (**G**) Results of CARBA-H and Carba NP upon incubation with clinical strains producing carbapenemases observed by the naked eye. Adapted with permission from ref. [36]. Copyright 2021, American Chemical Society.

Prof. Yang and the research team introduced a dual fluorogenic–colorimetric probe for rapid pan-carbapenemase detection, including the OXA type (Figure 8A) [36]. The detection of OXA-like carbapenemase was long considered a major challenge in rapid carbapenemase screening, partly because of the poor substrate–enzyme interaction and other mechanisms [37]. The researchers proposed that the existence of a 1*β*-methyl substituent in the carbapenem core stabilized the acyl-enzyme intermediate in OXA-48, thus retarding the hydrolysis of carbapenems. To offer experimental evidence, an alkyne-appended meropenem derivative named MERO-Alk was prepared to serve as a representative substrate (Figure 8B). The results obtained by in-gel fluorescent visualization strongly demonstrated that a stable covalent adduct was formed between MERO-Alk and recombinant OXA-48. Inspired by this, they constructed the carbapenemase probes from three critical building blocks, namely, a fluorophore (Resorufin), a carbapenem core, and a self-immolative spacer. CARBA-H and CARBA-Me, two probes without and with the 1*β*-substituent in the carbapenem core, respectively, were synthesized and evaluated. CARBA-H displayed much stronger fluorescence enhancement within 8 min than CB-1, a fluorogenic probe discussed before, when they both incubated with IMP-1 under the same condition. CARBA-H and CARBA-Me possessed better selectivity to carbapenemases over other *β*-lactamases, exhibiting significant fluorescence toward all carbapenemases, the concentration of which ranged from picomolar to sub-picomolar (Figure 8C–F). Reassuringly, CARBA-Me did not yield a notable response to OXA-48, which was consistent with the previous hypothesis. Encouraged by the results, clinical isolates including OXA-48, OXA-181, and OXA-232, as well as urine samples (culture-free) from patients with urinary tract infections (UTIs) were then used to further investigate the potential efficiency of the probe. As a result, CARBA-H demonstrated a distinct color change from pale orange to deep pink for all carbapenemases within 15 min under ambient light, and the fluorescence could also be seen under ultraviolet light to ensure accuracy. The color change and fluorescence turn-on shown upon interaction with OXA-type carbapenemases successfully proved the special ability of CARBA-H to realize broad-spectrum identification of carbapenemases, including carbapenemases from the OXA family. As expected, Carba NP provided false negative results on class D carbapenemases, even after 2 h of incubation in parallel experiments with CARBA-H, while CARBA-H provided 100% sensitivity and specificity (Figure 8G). All the experiments indicated that CARBA-H had better performance than existing experiments and probe designs, readily applied to quick carbapenemase screening.

## 5. Chemiluminescent Probe in Carbapenemase Detection

**Figure 9:**
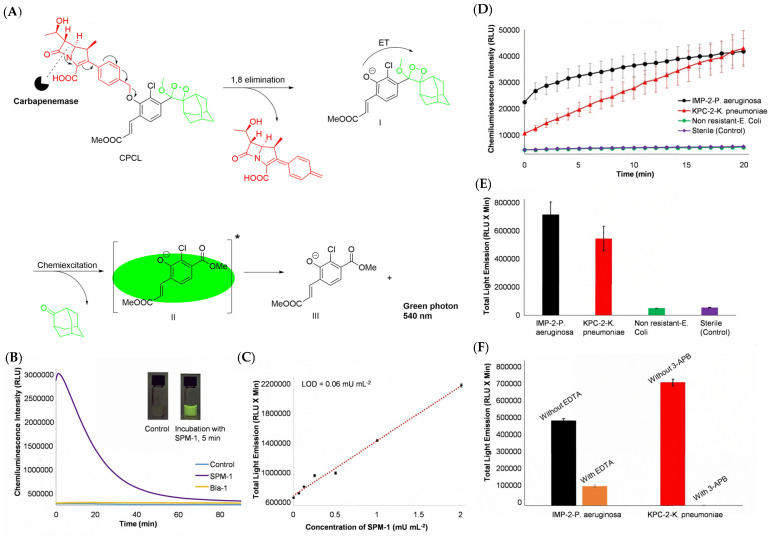
(**A**) The chemical excitation process of the carbapenemase detection probe, CPCL. (**B**) The chemiluminescence intensity of CPCL upon incubation with SPM-1 and Bla-1 compared with the control group. (**C**) The total light emission of CPCL according to the concentration of SPM-1. (**D**) Time-dependent chemiluminescence intensity of CPCL upon incubation with bacteria producing *β*-lactamase and non-CPB. (**E**) The total light emission of CPCL upon incubation with CPB and non-CPB. (**F**) The total light emission of CPCL upon incubation with CPB and non-CPB with and without the presence of inhibitors. Adapted with permission from ref. [38]. Copyright 2020, Wiley-VCH Verlag GmbH & Co. KGaA.

Inspired by the first and second generation carbapenemase fluorogenic probes developed by Rao’s and Xie’s groups, respectively, Shabat et al. reported the first carbapenemase chemiluminescent probe (CPCL) for the specific and sensitive detection of carbapenemase [38]. The unique chemical structure of CPCL consisted of a carbapenem core, which was connected to a dioxetane-based chemiluminescent luminophore through a 4-hydroxymethylphenyl self-immolative linker. As shown in Figure 9A, after enzymatic degradation of the carbapenemase core in the CPCL probe, a 1,8-elimination would spontaneously happen, thus producing phenolate-dioxetane I. Phenolate-dioxetane I rotted quickly into an excited benzoate ester II through chemical excitation. The energy was simultaneously released as a green photon as this invigorated benzoate ester decomposed to state III. After incubation with SPM-1 (Sao Paulo MBL), a broad-spectrum *β*-lactamase, and Bla-1 (a common carbapenemase), respectively, the ability of the CPCL probe to emit chemiluminescence was evaluated. As expected, CPCL released much stronger chemiluminescence for carbapenemase SPM-1 and reached a peak after 5 min with a low limit of detection (LOD), calculated to be 0.06 mU mL^−1^, while CPCL with Bla-1 provided only an insignificant signal (Figure 9B,C). Encouraged by the excellent specificity and sensitivity of CPCL, its ability to demonstrate the presence of live bacterial cells that produce carbapenemases was further confirmed by using the imipenem-resistant strains of *Pseudomonas aeruginosa* (harboring the IMP-2 carbapenemase gene) and *Klebsiella pneumoniae* (harboring the KPC-2 carbapenemase gene) as models. Figure 9D,E clearly show the significant light emission signal after pre-treating with imipenem-resistant strains of *Pseudomonas aeruginosa* and *Klebsiella pneumoniae*. In contrast, the light emission intensity in the carbapenemase-negative *E. coli* group and the sterile control group were both very low. The results obtained by the inhibition experiments with the addition of EDTA and 3-aminophenylboronic acid (3-APB) strongly supported the ability of CPCL to detect carbapenemase activity in test samples (Figure 9F). Likewise, clinically important carbapenemases, including VIM-15, KPC-1, and NMCA, were tested by CPCL, and satisfying results were observed, which demonstrated that CPCL could successfully select carbapenemases out of *β*-lactamases. Compared with fluorogenic probes that are easily distracted by background fluorogenic components, this chemiluminescent probe is less matrix-based and has a low price and simple operation, providing highly accurate results within 15 min.

## 6. Novel Intracellular Calorimetric Method in Carbapenemase Detection

**Figure 10:**
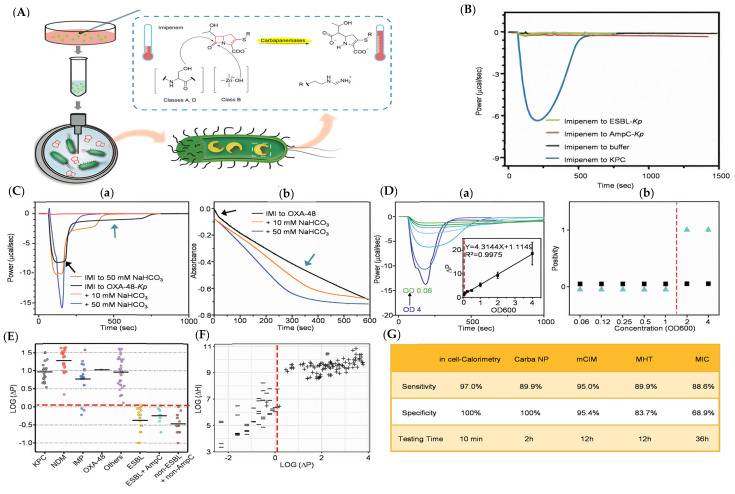
(**A**) Schematic outline of the experimental principle. (**B**) The heat change rate of hydrolyzation of imipenem by *β*-lactamase-producing bacteria and KPC. (**C**) The heat change rate and absorbance of imipenem hydrolyzed by OXA-48, in which the former presents a clear biphase kinetic profile. (**a**) Calorimetry method and (**b**) UV spectrophotometry test using OXA-48-*Kp* to determine the influence of carbon dioxide on the activity of OXA-48 to hydrolyze imipenem (black arrows: phase 1; cyan arrows: phase 2). (**D**) Examination of in-cell calorimetry method with Carba NP test for sensitivity by utilizing KPC-*Kp* at predetermined concentrations. (**a**) The linear relation between bacterial concentration and ΔP. (**b**) Positive results from the Carba NP test based on bacterial concentration (black squares: not positive; cyan triangles: positive). (**E**) Mapping of calorimetric results of 142 clinical strains catalyzing imipenem. (**F**) Dissemination of the log(ΔP) in light of *β*-lactamase type (“+”: carbapenemase positive; “-“: carbapenemase negative). (**G**) Differences among in-cell calorimetric test, Carba NP, mCIM, MIT, and MIC in sensitivity, specificity, and time. Adapted with permission from ref. [39]. Copyright 2018, Wiley Online Library.

Fluorogenic probes and chromogenic probes were designed to avoid signal interference, which is potentially caused by certain bacteria substrates or turbid bacterial suspension in spectrometry, LC-MS/MS, and MALDI-TOF MS, and they have resolved the inability to monitor real-time hydrolysis reactions [33]. However, the change in kinetic properties and the specificity of labeled substrates when encountering complex biological systems were unpredictable. Therefore, a novel method to detect carbapenemase by label-free natural substrates was utilized to observe enzyme activity in real-time [39]. Although hardly seen, monitoring the real-time heat change in biochemical reactions provided more precise and important data compared with traditional kinetic methods (Figure 10A) [40]. The researchers proved that the enthalpy and efficiency of *β*-lactam ring cleavage were distinguished significantly from background heat changes to guarantee the feasibility of the calorimetric method. Imipenem was chosen with and without the presence of carbapenemase inhibitors to confirm the enzymatic hydrolysis of carbapenem over time in living bacteria, the result of which was reflected by the heat change curve seen in Figure 10B. Surprisingly, a biphasic exothermic curve, which involved a quick exothermic surge and a slow exothermic decline, was observed in OXA-48-Kp, and this could be attributed to the carbamylation of a conserved lysine in some OXAs, formed by the reaction of carbon dioxide and the non-protonated ε-amino group of the lysine [41,42,43]. However, the effect of the biphase was imperceptible in UV assay, indicating the high sensitivity of the calorimetric approach and its potential to identify OXA carbapenemases (Figure 10C). A linear relationship was observed between bacterial concentration and heat change (ΔP), and the minimum detectable concentration of this method was much lower than that of the Carba NP test (Figure 10D). In direct comparison with the Carba NP test, the intracellular calorimetric test not only showed an extraordinary ability to generate real-time thermograms for OXA but also displayed at least two orders of magnitude more sensitivity in less than 10 min. In a small-scale screening test for CPE, a collection of 142 clinical Enterobacteriaceae isolates was included to assess the performance of the method, the result of which was 97% for sensitivity and 100% for specificity, while other assays, including Carba NP, mCIM, MHT, and MIC, all showed poorer results with longer testing times (Figure 10E–G). The only three false negative samples were IMP-1, IMP-4, and an unknown IMP-producing one, which all had low enzyme expressions. This natural substrate-based label-free method through imipenem catalysis was proven to be a simple, effective, and reliable tool to rapidly screen patients infected with CPB, and it also tracked the footprint and provided real-time information on the activity of all types of in-cell carbapenemase, including OXA-48-like carbapenemase. Even though they had applied carbapenem hydrolysis as an example of using heat change to reveal chemical reactions in living microbes, the idea was rather basic and was deemed relevant to a wide range of areas far beyond its immediate applications, which required a description of enzymatic activity in complex natural systems such as autoimmune cell reactions, tumor cell growth, and so on.

## 7. Conclusions and Perspectives

All the important small-molecule probes discussed throughout the review are summarized in Table 2 to allow clearer clarification. The emergence and spread of CPB have become a global health concern, necessitating the development of rapid and accurate detection methods to guide appropriate antibiotic therapy and prevent the further spread of antibiotic resistance [44]. Although carbapenem-derived small-molecule probes have made significant progress in recent years, most of the reported probes have good responsiveness to particular classes of carbapenemases only, and their sensitivity to the less-reactive OXA-48 and OXA-23 is still poor, which severely hinders the practical clinical application of these probes. In addition, probes from different technological routes also have their problems. For instance, fluorescent probes offer high sensitivity and specificity with rapidity, while cost-effective chromogenic methods are convenient, as they employ common reagents and do not require special instruments. However, these methods often have limited sensitivity in complex biological samples (sputum, blood, urine) and potential interference from endogenous substances, which lead to false positive/negative results. Modified chromogenic techniques have improved sensitivity compared with the original ones, making them valuable tools for the rapid screening of CPB. Yet, issues persist with signal stability and reproducibility in more diverse clinical settings. To overcome the above shortcomings, combining chemical biology tools for rational probe molecule designs in future research, to cover the entire range of carbapenemases, is necessary. Additionally, the compatibility of small-molecule probes with miniaturization and integration into portable devices makes them suitable for POCT, thereby reducing turnaround time; enhancing accessibility to diagnostic tools is the way forward [45]. Looking ahead, we call for the development of future detection methods to conquer the challenge of rapid carbapenemase subtyping with high accuracy, which ultimately advances the fight against antimicrobial resistance. Moreover, while ensuring the accuracy and speed of testing results, it is necessary to reduce the waste of medical resources and maximize the rational allocation of testing resources [46].

As the only rapid phenotypic detection method recommended by CLSI, the Carba NP test offers two significant advantages over most current clinical phenotypic methods: low cost and short detection time (2 h). However, compared with small-molecule probes, it is difficult to adopt in large-scale screening, as it involves more complex procedures, and its sensitivity and specificity are relatively poor. On the other hand, many small-molecule probes have high synthesis costs, require sophisticated instruments, and have poor accessibility. Additionally, they often suffer from reduced sensitivity to specific classes of carbapenemases such as class D (OXA-23, OXA-48) carbapenemases. Therefore, we believe that detection methods should be selected according to clinical needs in real practice. Small-molecule probes could be employed for screening CPB in large sample sets, followed by genotypic methods (such as PCR) to determine the specific bacterial genotypes. Moreover, small-molecule probes based on carbapenemase activity could, in theory, detect almost all classes and variants of carbapenemases without the need for genotypic identification. This could compensate for the limitation of genotypic methods, which are restricted to detecting known genes or proteins and may fail to detect novel or mutated carbapenemase strains. Therefore, small-molecule probes could serve as complementary tests to prevent missed detections in genotypic testing.

In the future, advanced probe designs will continue to improve the rapid detection of CPB. With the advent of artificial intelligence algorithms and the era of big data, using artificial intelligence models to predict the performance of probes in advance allows researchers to plan experimental designs more reasonably, save research and development costs, and accelerate the development process of probes for CPB detection. Nanotechnology-based detection methods, microfluidics, and biosensors may enhance sensitivity and allow for ultra-fast bacterial identification. Additionally, integrating these new techniques into portable diagnostic devices will improve accessibility in clinical and resource-limited settings.

## Figures and Tables

**Table 1:** Comparison of the common detection methods for carbapenemase in clinical practice.

Method	Subtype	Operability	Result	Time	Cost	Advantages	Limitations
Genetic test	Yes	Complex	Easy	70 min–4 h	High	Most direct and reliable to confirm the presence of carbapenemase and subtypes. PCR detects known types; sequencing discovers new mutated genes.	Expensive; Time-consuming; Professional and technical personnel.
MHT	No	Simple	False negative and false positive	24–48 h	Low	Simple; Practical	Poor detection of some AmpC/ultra-broad spectrum *β*-lactamases with membrane porin deletion.
Carba NP test	No	Simple	Easy	2 h	Low	Cost-effective; Rapid; CLSI recommended.	Needs special reagents, and some have a short validity period. Unable to detect specific types with low activity (e.g., OXA type).
DDST	Yes	Simple	Easy	24–48 h	Low	Low cost, Simple.	Cultured overnight; Poor timeliness.
mCIM test eCIM test	Yes	Complex	False negative and false positive	18–28 h	Low	Clinically used more; Dispense with special reagents or culture media.	Cultured overnight; Poor timeliness; Poor detection of OXA and IMP.
CDT	Yes	Simple	False positive	18–24 h	Low	Easy to execute with low cost and no need for specialized reagents.	Unable to accurately detect IMP-type and class D carbapenemases.
MHA	No	Simple	Easy	2–3 h	Low	Low cost; Precise result.	Need for special agents and equipment.

Abbreviations: MHT = modified hodge test; DDST = double disc synergy test; mCIM = modified carbapenemase inactivation method; eCIM = EDTA-modified carbapenem inactivation method; CDT = combined disk test; MHA = meropenem hydrolysis assay; CLSI = Clinical and Laboratory Standards Institute.

**Table 2:** Summary of structures of all the small-molecule probes discussed.

Category	Author	Year	Probe Structure
Chromogenic probes	Teethaisong, Y. et al. [22]	2019	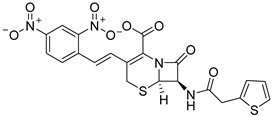
	Nordmann, P. et al. [23]	2020	
	Li, W. et al. [25]	2022	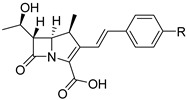
Fluorescent probes	Shi, H. et al. [30]	2014	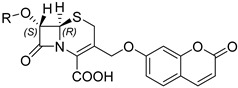
	Mao, W. et al. [33]	2017	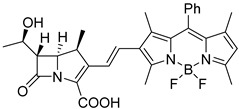
	Feng, Y. et al. [34]	2020	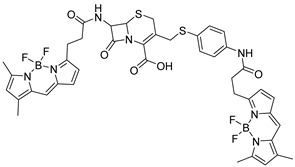
	Mao, W. et al. [35]	2019	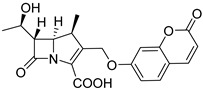
Dual fluorogenic–colorimetric probe	Ma, C. et al. [36]	2021	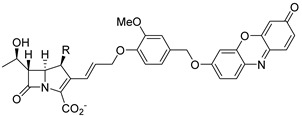
Chemiluminescent probe	Das, S. et al. [38]	2020	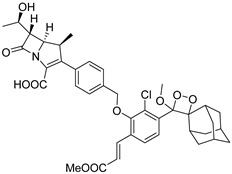
Calorimetric probe	Zhang, Y. et al. [39]	2018	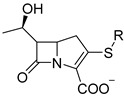

## Data Availability

The data that support the findings of this study are openly available.

## Abbreviations

The following abbreviations are used in this manuscript:

CPB	Carbapenemase-producing bacteria
MBLs	Metallo-*β*-lactamases
IMP	Imipenem enzyme-producing *β*-lactamase
VIM	Verona-integron encoded metallo-*β*-lactamase
NDM-1	New Delhi metallo-*β*-lactamase
PCR	Polymerase Chain Reaction
rt qRT-PCR	Real-time quantitative reverse transcriptase PCR
MHT	Modified Hodge test
E-test	Epsilometer Test
DDST	Double disc synergy test
LC-MS/MS	Liquid Chromatography–Tandem Mass Spectrometry
MALDI-TOF MS	Matrix-assisted Laser Desorption/Ionization Time-of-Flight Mass Spectrometry
CLSI	Clinical and Laboratory Standards Institute
CCS	Chromogenic carbapenem substrate
POCT	Point-of-care testing
CRE	Carbapenem-resistant Enterobacteriaceae
CPOs	Carbapenemase-producing organisms
UTI	Urinary tract infection
CPCL	Carbapenemase chemiluminescent probe
SPM-1	Sao Paulo metallo-*β*-lactamase
LOD	Limit of detection
PBS	Phosphate-buffered saline

## References

[1] Papp-Wallace K.M., Endimiani A., Taracila M.A., Bonomo R.A. (2011). Carbapenems: Past, present, and future. Antimicrob. Agents Chemother..

[2] Queenan A.M., Bush K. (2007). Carbapenemases: The versatile beta-lactamases. Clin. Microbiol. Rev..

[3] Nordmann P., Naas T., Poirel L. (2011). Global spread of Carbapenemase-producing Enterobacteriaceae. Emerg. Infect. Dis..

[4] Savard P., Perl T.M. (2014). Combating the spread of carbapenemases in Enterobacteriaceae: A battle that infection prevention should not lose. Clin. Microbiol. Infect..

[5] Bush K. (2018). Past and Present Perspectives on *β*-Lactamases. Antimicrob. Agents Chemother..

[6] Perez F., Bonomo R.A. (2019). Carbapenem-resistant Enterobacteriaceae: Global action required. Lancet Infect. Dis..

[7] Poirel L., Walsh T.R., Cuviilier V., Nordmann P. (2011). Multiplex PCR for detection of acquired carbapenemase genes. Diagn. Microbiol. Infect. Dis..

[8] Monteiro J., Widen R.H., Pignatari A.C.C., Kubasek C., Silbert S. (2012). Rapid detection of carbapenemase genes by multiplex real-time PCR. J. Antimicrob. Chemother..

[9] Avlami A., Bekris S., Ganteris G., Kraniotaki E., Malamou-Lada E., Orfanidou M., Paniara O., Pantazatou A., Papagiannitsis C.C., Platsouka E. (2010). Detection of metallo-*β*-lactamase genes in clinical specimens by a commercial multiplex PCR system. J. Microbiol. Methods.

[10] Meunier D., Hopkins K., Brown C., Cunningham N., Henderson K., Hopkins S., Mirfenderesky M., Woodford N. (2022). Commercial Assays for the Detection of Acquired Carbapenemases.

[11] Ramana K.V., Rao R., Sharada C.V., Kareem M., Reddy L.R., Mani M.R. (2013). Modified Hodge test: A useful and the low-cost phenotypic method for detection of carbapenemase producers in Enterobacteriaceae members. J. Nat. Sci. Biol. Med..

[12] Nordmann P., Poirel L., Dortet L. (2012). Rapid detection of carbapenemase-producing Enterobacteriaceae. Emerg. Infect. Dis..

[13] Zúñiga J., Cruz G., Pérez C., Tarajia M., LCRSP Microbiology Group (2016). The combined-disk boronic acid test as an accurate strategy for the detection of KPC carbapenemase in Central America. J. Infect. Dev. Ctries..

[14] Nachnani S., Scuteri A., Newman M.G., Avanessian A.B., Lomeli S.L. (1992). E-test: A new technique for antimicrobial susceptibility testing for periodontal microorganisms. J. Periodontol..

[15] Arakawa Y., Shibata N., Shibayama K., Kurokawa H., Yagi T., Fujiwara H., Goto M. (2000). Convenient test for screening metallo-beta-lactamase-producing gram-negative bacteria by using thiol compounds. J. Clin. Microbiol..

[16] Xia J., Gao J., Tang W. (2016). Nosocomial infection and its molecular mechanisms of antibiotic resistance. BioSci. Trends.

[17] Dixon B., Ahmed W.M., Felton T., Fowler S.J. (2022). Molecular phenotyping approaches for the detection and monitoring of carbapenem-resistant Enterobacteriaceae by mass spectrometry. J. Mass Spectrom. Adv. Clin. Lab..

[18] Li G., Ye Z., Zhang W., Chen N., Ye Y., Wang Y., Wu F., Wang K., Fan L. (2022). Rapid LC-MS/MS detection of different carbapenemase types in carbapenemase-producing Enterobacterales. Eur. J. Clin. Microbiol. Infect. Dis..

[19] Jing X., Hu Y., Wu T., Zhang X., Luo S., Wang W., Min X., Sun R., Zeng J. (2023). A rapid method for detecting and distinguishing metallo-*β*-lactamase-and serine carbapenemase-producing Enterobacteriales using MALDI-TOF MS. Front. Microbiol..

[20] Boehle K.E., Gilliand J., Wheeldon C.R., Holder A., Adkins J.A., Geiss B.J., Ryan E.P., Henry C.S. (2017). Utilizing Paper-Based Devices for Antimicrobial-Resistant Bacteria Detection. Angew. Chem. Int. Ed..

[21] (2019). Performance Standards for Antimicrobial Susceptibility Testing; Twenty-Fourth Informational Supplement.

[22] Teethaisong Y., Nakouti I., Evans K., Eumkeb G., Hobbs G. (2019). Nitro-Carba test, a novel and simple chromogenic phenotypic method for rapid screening of carbapenemase-producing Enterobacteriaceae. J. Glob. Antimicrob. Resist..

[23] Nordmann P., Sadek M., Demord A., Poirel L. (2020). NitroSpeed-Carba NP Test for Rapid Detection and Differentiation between Different Classes of Carbapenemases in Enterobacterales. J. Clin. Microbiol..

[24] Alizadeh N., Rezaee M.A., Kafil H.S., Barhaghi M.H.S., Memar M.Y., Milani M., Hasani A., Ghotaslou R. (2018). Detection of carbapenem-resistant Enterobacteriaceae by chromogenic screening media. J. Microbiol. Methods.

[25] Li W., Wang J., Li C., Zong Z., Zhao J., Gao H., Liu D. (2022). Achieving Ultrasensitive Chromogenic Probes for Rapid, Direct Detection of Carbapenemase-Producing Bacteria in Sputum. JACS Au.

[26] Gao X., Feng L., Deng R., Wang B., He Y., Zhang L., Luo D., Chen M., Chang K. (2024). Bottom-up DNA nanostructure-based paper as point-of-care diagnostic: From method to device. Interdiscip. Med..

[27] Bialvaei A.Z., Kafil H.S., Asgharzadeh M., Memar M.Y., Yousefi M. (2016). Current methods for the identification of carbapenemases. J. Chemother..

[28] Kong Y., Jiang Q., Zhang F., Yang Y. (2023). Small Molecular Fluorescent Probes: Application Progress of Specific Bacteria Detection and Antibacterial Phototherapy. Chem. Asian J..

[29] Kim J., Kim Y., Abdelazem A.Z., Kim H.J., Choo H., Kim H.S., Kim J.O., Park Y.J., Min S.J. (2020). Development of carbapenem-based fluorogenic probes for the clinical screening of carbapenemase-producing bacteria. Bioorg. Chem..

[30] Shi H., Cheng Y., Lee K.H., Luo R.F., Banaei N., Rao J. (2014). Engineering the stereochemistry of cephalosporin for specific detection of pathogenic carbapenemase-expressing bacteria. Angew. Chem. Int. Ed..

[31] El-Gamal M.I., Brahim I., Hisham N., Aladdin R., Mohammed H., Bahaaeldin A. (2017). Recent updates of carbapenem antibiotics. Eur. J. Med. Chem..

[32] Walsh T.R., Toleman M.A., Poirel L., Nordmann P. (2005). Metallo-beta-lactamases: The quiet before the storm?. Clin. Microbiol. Rev..

[33] Mao W., Xia L., Xie H. (2017). Detection of Carbapenemase-Producing Organisms with a Carbapenem-Based Fluorogenic Probe. Angew. Chem. Int. Ed..

[34] Feng Y., Palanisami A., Kuriakose J., Pigula M., Ashraf S., Hasan T. (2020). Novel Rapid Test for Detecting Carbapenemase. Emerg. Infect. Dis..

[35] Mao W., Wang Y., Qian X., Xia L., Xie H. (2019). A Carbapenem-Based Off-On Fluorescent Probe for Specific Detection of Metallo-*β*-Lactamase Activities. ChemBioChem.

[36] Ma C., Ng K.K., Yam B.H., Ho P., Kao R.Y., Yang D. (2021). Rapid Broad Spectrum Detection of Carbapenemases with a Dual Fluorogenic-Colorimetric Probe. J. Am. Chem. Soc..

[37] Pitout J.D.D., Peirano G., Kock M.M., Strydom K., Matsumura Y. (2019). The Global Ascendency of OXA-48-Type Carbapenemases. Clin. Microbiol. Rev..

[38] Das S., Ihssen J., Wick L., Spitz U., Shabat D. (2020). Chemiluminescent Carbapenem-Based Molecular Probe for Detection of Carbapenemase Activity in Live Bacteria. Chem. Eur. J..

[39] Zhang Y., Lei J., He Y., Yang J., Wang W., Wasey A., Xu J., Lin Y., Fan H., Jing G. (2018). Label-Free Visualization of Carbapenemase Activity in Living Bacteria. Angew. Chem. Int. Ed. Engl..

[40] Mazzei L., Ciurli S., Zambelli B. (2014). Hot biological catalysis: Isothermal titration calorimetry to characterize enzymatic reactions. J. Vis. Exp..

[41] Vercheval L., Bauvois C., Paolo A.D., Borel F., Ferrer J., Sauvage E., Matagne A., Frère J., Charlier P., Galleni M. (2010). Three factors that modulate the activity of class D *β*-lactamases and interfere with the post-translational carboxylation of Lys70. Biochem. J..

[42] Verma V., Testero S.A., Amini K., Wie W., Liu J., Balachandran N., Monoharan T., Stynes S., Kotra L.P., Golemi-Kotra D. (2011). Hydrolytic mechanism of OXA-58 enzyme, a carbapenem-hydrolyzing class D *β*-lactamase from Acinetobacter baumannii. J. Biol. Chem..

[43] Golemi D., Maveyraud L., Vakulenko S., Samama J.P., Mobashery S. (2001). Critical involvement of a carbamylated lysine in catalytic function of class D beta-lactamases. Proc. Natl. Acad. Sci. USA.

[44] Bonomo R.A., Burd E.M., Conly J., Limbago B.M., Poirel L., Segre J.A., Westblade L.F. (2018). Carbapenemase-Producing Organisms: A Global Scourge. Clin. Infect. Dis..

[45] McConnell P., Einav S. (2023). Resource allocation. Curr. Opin. Anaesthesiol..

[46] Liang D., Wang Y., Qian K. (2023). Nanozymes: Applications in clinical biomarker detection. Interdiscip. Med..

